# Intravenous application of tranexamic acid in intramedullary nailing for the treatment of geriatric intertrochanteric fractures: a systematic review and meta-analysis

**DOI:** 10.1186/s12891-023-06725-y

**Published:** 2023-07-27

**Authors:** Jiakai Zhang, Xiaoyuan Fan, Yi Zheng, Junlong Wu, Xinhua Yuan

**Affiliations:** 1 Department of Orthopedics, Ningbo No.2 Hospital, Ningbo, 315000 Zhejiang China; 2 Department of Gastroenterology, The Affiliated People’s Hospital of Ningbo University, Ningbo, 315040 Zhejiang China

**Keywords:** Intravenous application, Tranexamic acid, Intertrochanteric fracture, Intramedullary nailing

## Abstract

**Background:**

Meta-analyses on the use of tranexamic acid (TXA) in intertrochanteric fractures have shown inconsistent results due to variations in inclusion criteria and clinical heterogeneity. To address these limitations, we conducted a rigorous analysis of recent randomized controlled trials (RCTs) with strict inclusion criteria. The aim of this study was to objectively evaluate the effects and safety of intravenous TXA administration in the treatment of geriatric intertrochanteric femoral fractures with intramedullary nailing.

**Methods:**

PubMed, Embase, and the Cochrane Library were searched for RCTs published from the database inception to August 2022. The date of total blood loss (TBL), intra-operative blood loss (IBL), hidden blood loss (HBL), transfusion rate, transfusion units, thromboembolic events, and mortality were extracted. Review Manager 5.3 was used for the analysis.

**Results:**

A total of six RCTs involving 689 patients were included. Meta-analyses indicated that TXA can significantly reduce TBL (WMD = -232.82; 95% CI -312.81 to -152.84; *p* < 0.00001), IBL (WMD = -36.33; 95% CI -51.38 to -21.28; *p* < 0.00001), HBL (WMD = -189.23; 95% CI -274.92 to -103.54; *p* < 0.0001), transfusion rate (RR = 0.53; 95% CI 0.33 to 0.85; *p* = 0.008), and transfusion units (WMD = -0.58; 95% CI -0.75 to -0.41; *p* < 0.01). No increase in thromboembolic events rate (RR = 0.75; 95% CI 0.38 to 1.50; *p* = 0.42) and mortality (RR = 1.36; 95% CI 0.61 to 3.04; *p* = 0.45) was observed.

**Conclusions:**

Our meta-analysis provides robust evidence supporting the efficacy and safety of intravenous TXA administration in treating geriatric intertrochanteric femoral fractures with intramedullary nailing. TXA significantly reduces blood loss and transfusion requirements without increasing the risk of thromboembolic events or mortality.

**Supplementary Information:**

The online version contains supplementary material available at 10.1186/s12891-023-06725-y.

## Introduction

Intertrochanteric fractures of the femur are common among individuals aged 60 years and above [1]. The global incidence is expected to reach 6,300,000 per year by 2050 [2, 3]. Surgical intervention is often recommended for intertrochanteric fractures, as conservative treatment can often result in a 30% 1-year mortality rate due to various complications [4, 5]. These fractures frequently result in significant blood loss at the fracture site, which, in combination with perioperative blood loss, increases the risk of perioperative anemia [3]. Anemia can complicate a patient’s condition, particularly when there are preexisting risks, and it can hinder the recovery process [6, 7]. Allogeneic blood transfusions are often required to treat severe anemia but can carry the risk of transfusion reactions and infections [8]. Therefore, reducing perioperative blood loss is crucial for minimizing complications and improving prognosis. Intramedullary nailing is a popular technique due to its minimally invasive nature, reliable fixation, and low intraoperative blood loss (IBL) [9–11]. However, it is worth noting that hidden blood loss (HBL) in intertrochanteric fractures is significantly higher than IBL [12, 13], emphasizing the importance of reducing HBL to determine a patient’s prognosis for femoral intertrochanteric fractures.

Tranexamic acid (TXA) is a synthetic antifibrinolytic drug [14]. Numerous studies have shown that tranexamic acid administered intravenously during hip and knee replacement reduces blood loss and lowers transfusion rates without raising risk of complications [15, 16]. In recent years, TXA has also been used in intramedullary fixation for the treatment of intertrochanteric fractures, as indicated by several studies [17–20]. Based on the findings of these studies, several meta-analyses have been conducted [21–23]. However, the clinical heterogeneity resulting from variations in TXA administration methods and doses, fracture types, surgical options, and outcome evaluation methods has compromised the credibility of the conclusions.

To better assess the effectiveness and safety of intravenous administration of TXA in the treatment of geriatric intertrochanteric fractures with intramedullary nailing, we used stringent inclusion and exclusion criteria and thorough analysis of the most recent published randomized controlled trials (RCTs).

## Materials and methods

This systematic review protocol has been registered on PROSPERO as CRD42023389130. This meta-analysis was conducted following the Preferred Reporting Items for Systematic Reviews and Meta-Analyses (PRISMA) Statement.

### Search strategy

The electronic databases of PubMed, Embase, and the Cochrane library were searched from the inception of the database to August 2022, only English studies were included. Two reviewers independently conducted literature searches using the following keywords: “tranexamic acid”, “TXA”, “intertrochanteric fracture”, “trochanteric fracture”, “hip fracture”, “intramedullary nailing”, “IMN”, “proximal femoral nail anti-rotation”, “PFNA”. Additionally, for more appropriate studies, the references of previously published literature were manually searched.

### Eligibility criteria

The inclusion criteria for this study were as follows:

1. Intertrochanteric fracture patients (age ≥ 60 years) undergoing proximal femoral intramedullary nail surgery.
2. Intravenous administration of TXA in the experimental group. No TXA administration in the control group, with placebo or saline.
3. RCTs only.
4. The clinical outcome data were total blood loss (TBL), IBL, HBL, transfusion rate, transfusion units, mortality, and thromboembolic events, which included symptomatic deep venous thrombosis (DVT), pulmonary embolism (PE), myocardial infarctions and strokes. Studies had to include at least one clinical outcome statistic.

The exclusion criteria were as follows:

1. Patients with open or multiple fractures.
2. Retrospective study, review articles, case series, and case reports were excluded.
3. Did not contain the outcome indicators of inclusion criteria.

### Data extraction

Two reviewers independently extracted data according to the inclusion and exclusion criteria. Any disagreement on data extraction was resolved by the third reviewer. The demographic characteristics and clinical outcome were extracted as follows: first author, publication year, region, case number, age, gender, fixation method, thromboprophylaxis drugs, interventions, transfusion criteria, TBL, IBL, HBL, transfusion rate, transfusion units, follow-up, thromboembolic events, and mortality.

### Quality assessment

Using the Cochrane Collaboration’s risk of bias tool [24], two reviewers independently evaluated the methodological quality. A discussion or consultation with the third reviewer helped to resolve disagreements.

### Statistical analysis

Review Manager 5.3 was used for the analysis. *P* < 0.05 was considered statistically significant. Relative risk (RR) and 95% confidence interval (CI) were used for dichotomous outcomes; mean difference (MD) and 95% CI were used for continuous outcomes. Statistical heterogeneity for all enrolled studies was evaluated using the Chi-square test and *I*^2^ statistic. Statistical heterogeneity was classified into three categories: high (*I*^2^ ≥ 50%), moderate (25% ≤ *I*^2^ < 50%), and low (*I*^2^ < 25%). If the *p* value of heterogeneity was less than 0.1, heterogeneity would exist. The random effects model was performed when *I*^2^ ≥ 50%, Otherwise, the fixed-effects model was chosen.

## Results

### Included studies

**Fig. 1:**
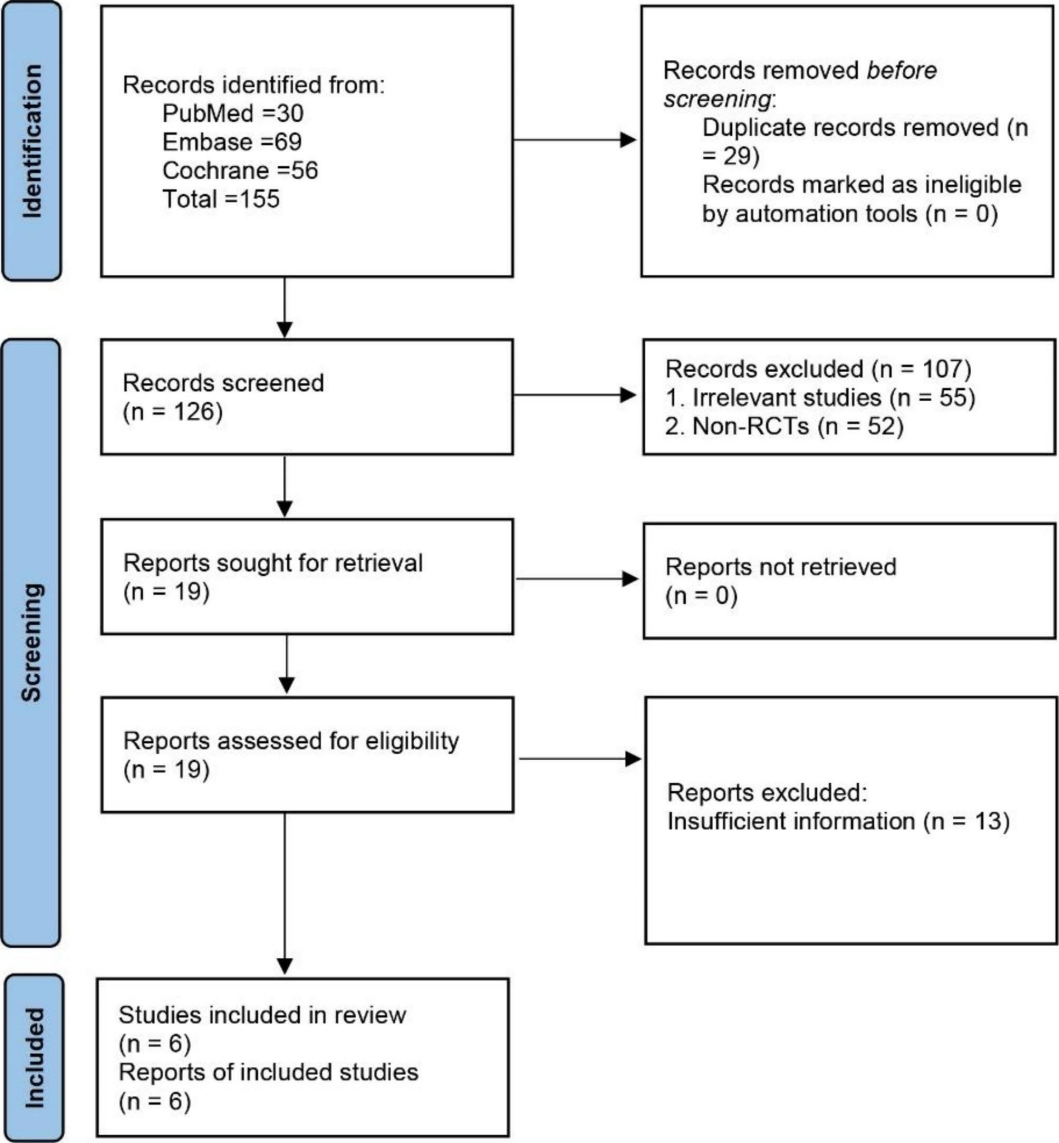
PRISMA flowchart of study selection process

In the initial search, a total of 155 potentially relevant publications were found. After removing duplicate studies and filtering titles and abstracts, 19 studies remained. Thirteen studies were excluded because they did not meet one or more of our selection criteria. Finally, 6 studies [17–20, 25, 26] were included in our quantitative synthesis (Fig. 1).

### Study characteristics and quality assessment

**Fig. 2:**
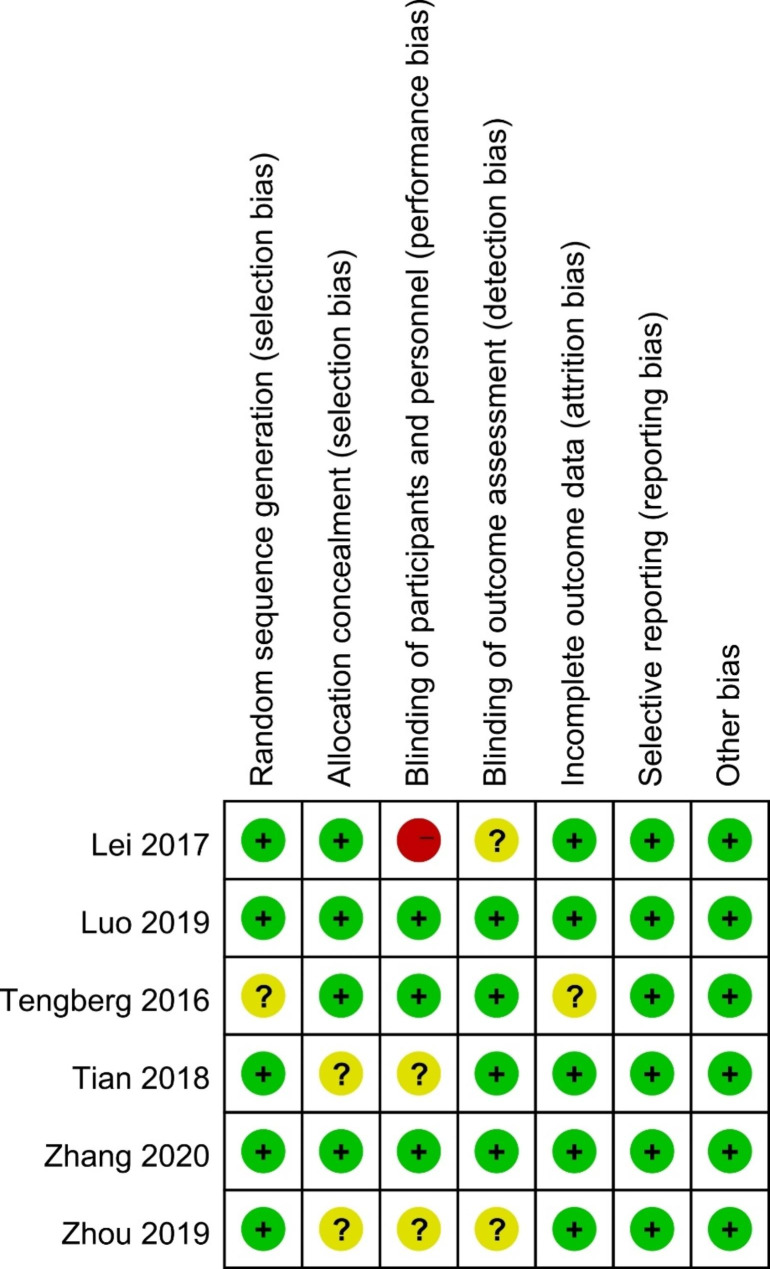
Risk of bias summary

The main characteristics of the included studies are shown in Table 1. All studies were published between 2016 and 2021. A total of 689 patients were enrolled in the analysis, including 340 patients in the TXA group and 349 patients in the control group. All patients enrolled were diagnosed with intertrochanteric fractures and treated by closed fracture reduction and fixation with intramedullary nails. There was no significant difference in baseline between the two groups. All studies included were high-quality RCTs. The risk of bias summary for each of the included studies was shown in Fig. 2.

**Table 1:** The main characteristics of the included participants

Study (Year)	Region	Patients (T/C)	Age (years, T/C)	Female gender (T/C)	Fixation method	Thromboprophylaxis drugs	Transfusion criteria	Intervention	Control	Follow up (months)
Lei 2017	China	37/40	77.8/79.18	32/33	PFNA	LMWH	Hb < 9 g/dl	1 g IV TXA before surgery	200 mL saline	1
Luo 2019	China	44/46	75.1/76.1	21/26	PFNA	LMWH	Hb < 8 g/dl	2 doses of 15 mg/kg IV TXA before surgery and 3 h later	100 mL saline	1.5
Tengberg 2016	Denmark	33/39	79.8/75	26/25	IMHS	LMWH	Hb < 9.67 g/dl	1 g IV TXA before surgery, 3 g post-operative 24 h infusion	Placebo	3
Tian 2018	China	50/50	77.74/79.25	31/36	PFNA	NR	Hb < 9 g/dl	2 doses of 10 mg/kg IV TXA 10 min preoperatively and 5 h postoperatively	No TXA administration	NR
Zhang 2020	China	61/61	79.11/76.07	33/27	PFNA	LMWH	Hb < 7 g/dl or symptomatic anemia with an Hb level of 7–10 g/dl	1 g of IV-TXA before incision and 3 h later	100 mL saline	3
Zhou 2019	China	50/50	75.10/77.82	35/28	PFNA	LMWH	Hb < 7 g/dl	1 g IV TXA before surgery	placebo	1

Note: PFNA Proximal femoral nail anti-rotation, LMWH Low molecular weight heparin, IMHS Intramedullary hip screw, IV Intravenous injection, NR No record

### Results of meta-analysis

#### TBL

**Fig. 3:**
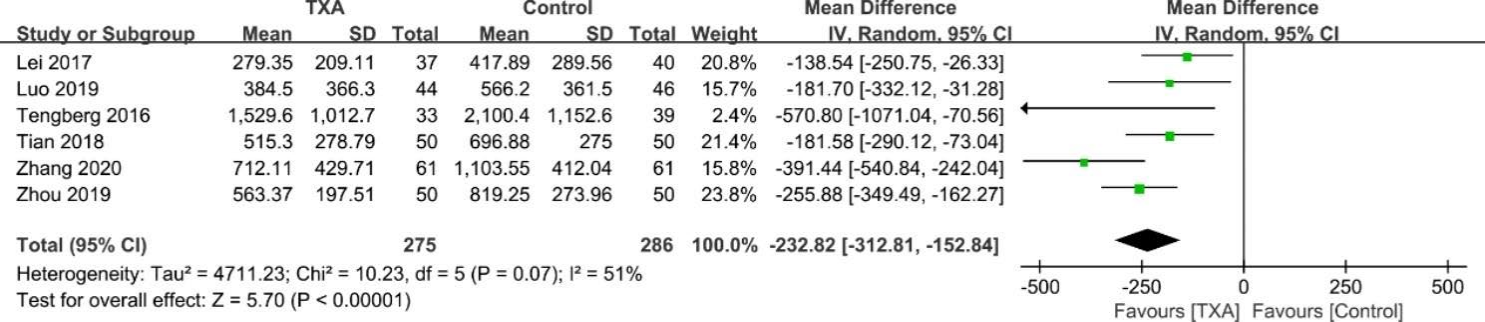
Forest plot for total blood loss

Six studies [17–20, 25, 26] reported the results of TBL. Significant heterogeneity was found in the pooled outcomes, so a random effects model was used (χ^2^ = 10.23, df = 5, *I*^2^ = 51%, *p* = 0.07). The pooled results indicated that there was a significant difference between the two groups (WMD = -232.82; 95% CI -312.81 to -152.84; *p* < 0.00001; Fig. 3). A random effects model was adopted.

#### IBL

**Fig. 4:**
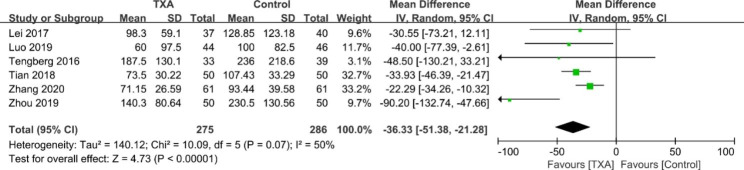
Forest plot for intraoperative blood loss

The results of IBL were documented in six studies [17–20, 25, 26]. A random effects model was adopted because the pooled data had substantial heterogeneity (χ^2^ = 10.09, df = 5, *I*^2^ = 50%, *p* = 0.07). The combined data showed that the TXA group had significantly less IBL than the control group (WMD = -36.33; 95% CI -51.38 to -21.28; *p* < 0.00001; Fig. 4).

#### HBL

**Fig. 5:**
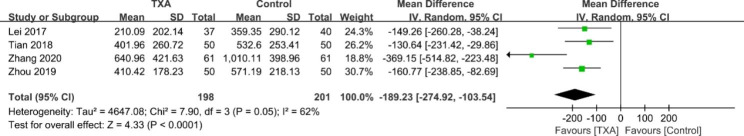
Forest plot for hidden blood loss

HBL was reported in four studies [17, 19, 20, 26]. A random effects model was chosen since the pooled results had significant heterogeneity (χ^2^ = 7.90, df = 3, *I*^2^ = 62%, *p* = 0.05). According to the pooled data, TXA considerably decreased HBL (WMD = -189.23; 95% CI -274.92 to -103.54; *p* < 0.0001; Fig. 5).

#### Transfusion rate

**Fig. 6:**
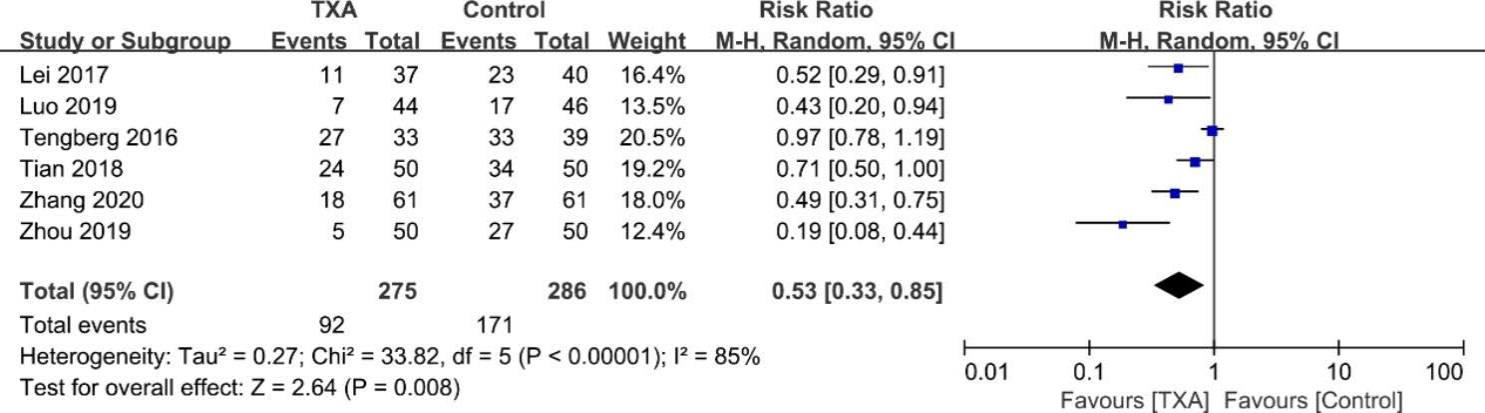
Forest plot for transfusion rate of allogenic blood

The transfusion rate was mentioned in all six investigations [17–20, 25, 26]. The pooled results showed substantial heterogeneity (χ^2^ = 33.82, df = 5, *I*^2^ = 85%, *p* < 0.00001), and therefore, a random effects model was used. The analysis demonstrated that TXA significantly decreased the transfusion rate (RR = 0.53; 95% CI 0.33 to 0.85; *p* = 0.008, Fig. 6).

#### Transfusion units

**Fig. 7:**
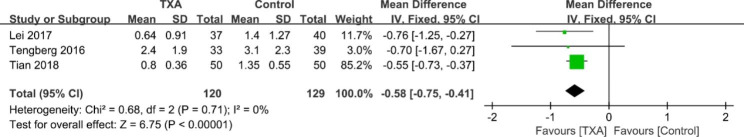
Forest plot for transfused RBC units per patient

Three studies [17, 25, 26] provided data on transfusion units. The pooled results showed the use of TXA reduced the transfusion units by 0.44 units per patient with significant difference (WMD = -0.58; 95% CI -0.75 to -0.41; *p* < 0.01; *I*^2^ = 0%, Fig. 7).

#### Thromboembolic events

**Fig. 8:**
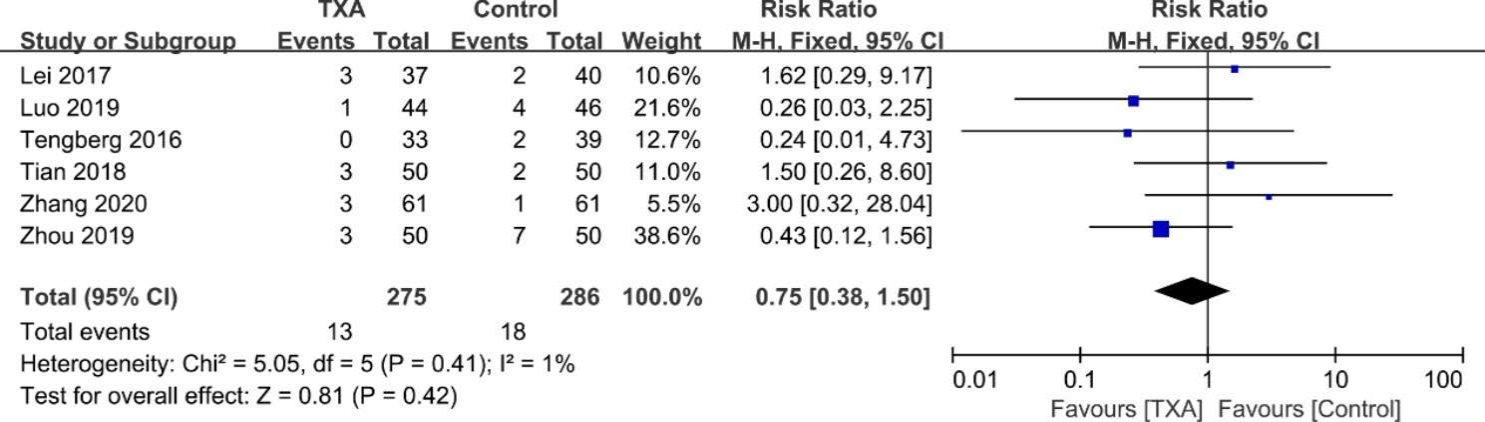
Forest plot for thromboembolic events rate

The pooled results of six studies [17–20, 25, 26] showed no significant difference in the postoperative occurrence of thromboembolic events between TXA and control groups (RR = 0.75; 95% CI 0.38 to 1.50; *p* = 0.42, *I*^2^ = 1%, Fig. 8).

#### Mortality

**Fig. 9:**
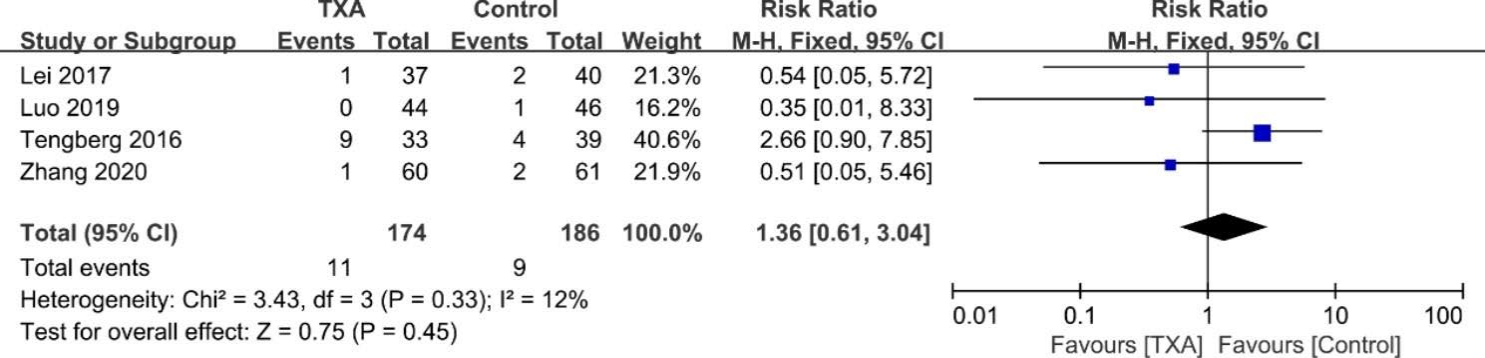
Forest plot for mortality

Four studies [17–19, 25] reported mortality in 90 days after the operation. We found that there was no significant difference between the two groups (RR = 1.36; 95% CI 0.61 to 3.04; *p* = 0.45, *I*^2^ = 12%, Fig. 9).

#### Publication bias

**Fig. 10:**
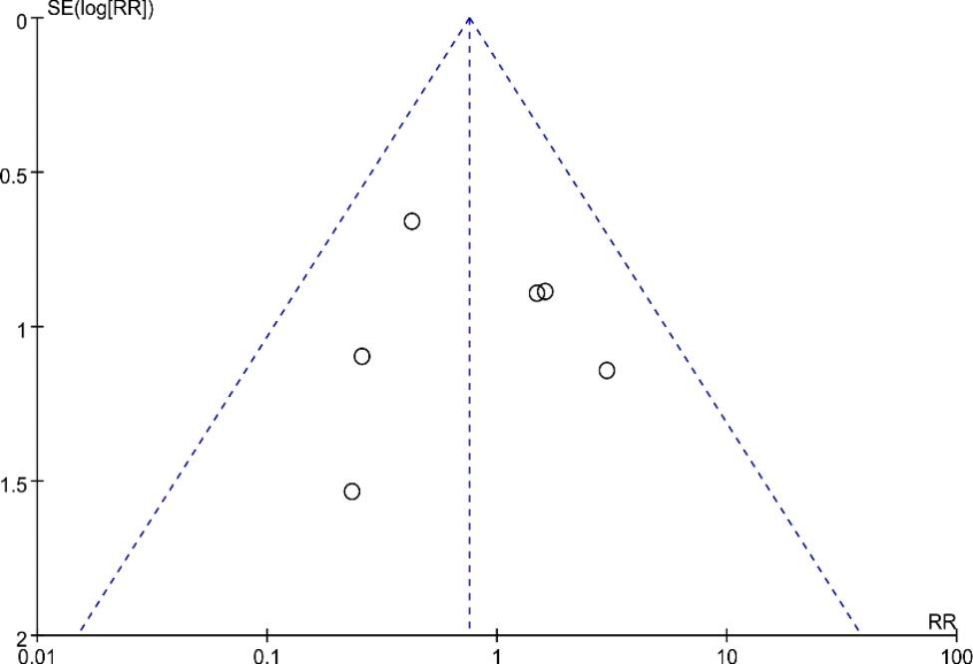
Funnel plot of thrombotic events

The funnel plot analysis of thrombotic events revealed a generally symmetrical distribution of scattered data points, indicating a minimal presence of publication bias among the studies included in the analysis (Fig. 10).

## Discussion

In recent years, several meta-analyses have been published on the use of TXA in hip fractures. Luo [21] conducted an analysis of 5 RCTs with 540 cases, evaluating the role and safety of TXA in intramedullary nailing for the treatment of geriatric intertrochanteric fractures. The study concluded that TXA significantly reduced TBL, IBL, and HBL, while also decreasing transfusion ratios and units, without increasing the risk of thrombosis. Similarly, Xing’s meta-analysis [27] included 5 RCTs with 539 cases and examined the role of TXA in intramedullary nailing of the proximal femur for geriatric intertrochanteric fractures. TXA was found to effectively reduce TBL, IBL, HBL, transfusion rates, hospital stay, and wound hematoma rates, without increasing operative time, postoperative mortality, thrombotic events, wound infections, cerebrovascular accidents, respiratory infections, or renal failure. Another meta-analysis by Yu [23] investigated the use of TXA in geriatric intertrochanteric fractures, encompassing 11 RCTs with 1202 cases. The analysis demonstrated that TXA reduced IBL, HBL, TBL, hospital stay, transfusion ratios, and the risk of wound complications, while also resulting in higher hemoglobin levels at postoperative days 1, 2, and 3. There were no significant differences in thrombotic risk or mortality. Additionally, a meta-analysis by Masouros [22] included 10 randomized controlled studies involving 1123 cases to evaluate the role of intravenous TXA in hip fractures among the elderly. The results indicated that TXA effectively reduced TBL, transfusion ratios, and transfusion units, without increasing the risk of thrombotic events. Moreover, the results of a single dose of 15 mg/kg were comparable to multiple doses.

However, it is important to note that there was significant clinical heterogeneity among the studies included in the aforementioned meta-analyses. These studies employed different methods of TXA application, had varying patient ages, fracture types, and surgical options. To address the issue of clinical heterogeneity and enhance result reliability, this study specifically focused on the intravenous application of TXA. The effectiveness of topical application for intertrochanteric fractures remains uncertain [28], and some studies suggest that it does not lead to reduced blood loss [29, 30]. Therefore, it would be inappropriate to combine studies that employ different application methods. Furthermore, this study focused on geriatric intertrochanteric fractures and excluded femoral neck fractures, as the surgical options for the two types of fractures are distinct and not comparable. The study also restricted the surgical option to proximal femoral intramedullary nailing, which is more commonly used, associated with shorter operative times, reduced blood loss, fewer complications, and improved functionality [9, 10]. Ultimately, six high-quality randomized controlled studies [17–20, 25, 26] were included in our study. The meta-analysis results demonstrated that intravenous TXA administration during intramedullary nailing for geriatric intertrochanteric fractures effectively reduced TBL, IBL, and HBL, decreased transfusion rates and units, and did not increase the risk of thrombotic events or mortality, consistent with the findings of previous studies [21–23, 27].

As the concept of enhanced recovery gains prominence, the significance of blood management becomes increasingly evident [31]. Adequate blood management measures are crucial for achieving early and rapid recovery in patients, particularly among the elderly. This study utilized TBL, IBL, and HBL as primary observations, employing the Nadler [32] and Gross [33] formulas for blood loss calculations as commonly mentioned in the literature. TBL calculations depend on blood volume, preoperative and postoperative hematocrit levels, while IBL calculations involve intraoperative suction flow and blood volume on the gauze. HBL is calculated by subtracting IBL from TBL. If intraoperative or postoperative blood transfusion occurred, the transfusion volume was added to TBL. This formula was utilized consistently across all studies. The results of our study indicated a statistically significant decrease in TBL (approximately 232.82 ml, *p* < 0.00001), IBL (approximately 36.33 ml, *p* < 0.00001), and HBL (approximately 189.23 ml, *p* < 0.0001) in the TXA group compared to the control group. However, significant heterogeneity was observed in the three pooled analyses, which is consistent with previous meta-analyses [23]. Using the leave-one-out technique, the *I*^2^ value for heterogeneity in the TBL results decreased from 51 to 17%. Exclusion of Zhang’s study [19] led to a reduction in the *I*^2^ value for HBL results from 62 to 0%. One possible source of heterogeneity could be the variation in TBL and HBL calculation strategies. Although the Nadler and Gross formulas were used in all studies, there were differences in specific calculation methods. Furthermore, variations in blood test timing were observed, with some studies conducting preoperative tests upon admission [19, 25] or one day before surgery [17, 18, 20], while others performed them on the morning of the surgery [26]. Postoperative blood data were also measured at different times, with some studies using the lowest values on postoperative days 1 and 3 [17, 19–21, 25], and others using values from day 2 [26]. Differences in measurement timing may introduce bias in TBL calculations, contributing to heterogeneity in the meta-analysis results. Additionally, excluding Zhou’s study [20] led to a decrease in the *I*^2^ value for IBL results from 50 to 0%. IBL calculation is unrelated to blood tests, and the methodology employed was essentially the same. Despite attempts at subgroup analysis to identify the cause of heterogeneity, it remained unresolved. Nonetheless, even after excluding the study with significant heterogeneity, the final statistical results remained significantly different.

Reducing transfusion rates is another important aspect of assessing the bleeding-reducing effect of TXA. Our study found that the perioperative transfusion rate was 33.45% in the TXA group and 57.79% in the control group, demonstrating a statistically significant difference (RR = 0.53; 95% CI 0.33 to 0.85; *p* = 0.008). This indicates that the TXA group had a 47% lower transfusion risk compared to the control group. Moreover, transfusion units decreased by an average of 0.58 units per patient (WMD = -0.58; 95% CI -0.75 to -0.41; *p* < 0.01). However, notable heterogeneity was observed in the combined transfusion rate data (χ2 = 33.82, df = 5, *I*^2^ = 85%, *p* < 0.00001), and heterogeneity persisted even after employing the leave-one-out technique. The variation in transfusion strategies across studies was identified as a contributing factor.

Although there is evidence supporting the effectiveness of TXA in reducing blood loss, concerns have been raised regarding increased thrombotic events and mortality. Studies by Zufferey [34] and Schiavone [35] reported three times higher thrombotic event and mortality rates in the TXA group compared to the control group, but without statistical significance. Franchini’s meta-analysis [36] investigating the safety of intravenous TXA in orthopedic surgery included 73 RCTs with 4174 cases in the TXA group and 2779 cases in the control group. The pooled analysis concluded that the risk of thrombosis between the TXA and control groups was comparable and not statistically different. A large retrospective study by Zak [37] involving 26,808 patients with coronary artery disease or coronary stenting who underwent total knee or total hip replacement concluded that TXA was safe and did not increase the risk of thrombosis during an 8-year follow-up period. Furthermore, TXA applied to intertrochanteric fractures in patients with a high risk of thrombosis, as determined by a scoring scale, demonstrated an excellent safety profile in a retrospective study by Porter [38]. There were no statistically significant differences in thrombotic events or mortality compared to the control group. These findings are consistent with our study results.

Nevertheless, our study has several limitations. Despite efforts to strictly limit the study criteria to reduce clinical heterogeneity, some differences in research methods cannot be entirely eliminated. Factors such as preoperative waiting time, blood test timing, TXA dosage, use of low-molecular-weight heparin, and transfusion strategies may influence the study results. Additionally, the detailed calculation of blood loss varies among studies, making the data less comparable, which is a limitation considering blood loss was the primary outcome of this study. Furthermore, the number of included studies was relatively small, and the total number of cases was limited. Lastly, some studies had a follow-up period of only one month, lacking long-term follow-up data. Despite these limitations, our study included the most recent high-quality RCTs, and there was strong statistical evidence supporting the study results.

## Conclusion

In conclusion, the intravenous application of TXA in geriatric patients undergoing intramedullary nailing for the treatment of intertrochanteric fractures is both safe and effective in reducing TBL, IBL, and HBL. It also decreases transfusion rates and volumes without increasing the risk of thrombotic events or mortality.

## Electronic supplementary material

Below is the link to the electronic supplementary material.

Supplementary Material 1

## Data Availability

The datasets used and/or analyzed during the current study are available from the corresponding author on reasonable request.

## Abbreviations

TXA: Tranexamic acid
RCT: Randomized controlled trial
IBL: Intra-operative blood loss
HBL: Hidden blood loss
TBL: Total blood loss
VBL: Visible blood loss
DVT: Deep venous thrombosis
PE: Pulmonary embolism
RR: Relative risk, CI:Confidence interval
MD: Mean difference
PRISMA: Preferred Reporting Items for Systematic Reviews and Meta-Analyses
