# Supplementary material for: Intravenous application of tranexamic acid in intramedullary nailing for the treatment of geriatric intertrochanteric fractures: a systematic review and meta-analysis
Source: BMC Musculoskelet Disord. 2023 Jul 27;24:614. doi: 10.1186/s12891-023-06725-y (PMC10373423; doi:10.1186/s12891-023-06725-y)
Supplement: Supplementary file 1 — Supplementary Material 1 [file 12891_2023_6725_MOESM1_ESM.pdf]

Supplementary Material

Search Strategies:

Pubmed

("Hip Fractures"[MeSH Terms] OR ("Hip Fractures"[MeSH Terms] OR ("hip"[All Fields] AND "fractures"[All Fields]) OR "Hip Fractures"[All Fields] OR ("intertrochanteric"[All Fields] AND "fractures"[All Fields]) OR "intertrochanteric fractures"[All Fields] OR ("Hip Fractures"[MeSH Terms] OR ("hip"[All Fields] AND "fractures"[All Fields]) OR "Hip Fractures"[All Fields] OR ("trochanteric"[All Fields] AND "fracture"[All Fields]) OR "trochanteric fracture"[All Fields]) OR ("Hip Fractures"[MeSH Terms] OR ("hip"[All Fields] AND "fractures"[All Fields]) OR "Hip Fractures"[All Fields] OR ("hip"[All Fields] AND "fracture"[All Fields]) OR "hip fracture"[All Fields]))) AND ("Tranexamic Acid"[MeSH Terms] OR ("tranexamic"[All Fields] AND "acid"[All Fields]) OR "Tranexamic Acid"[All Fields] OR "TXA"[All Fields] OR "Tranexamic Acid"[MeSH Terms]) AND ("fracture fixation, intramedullary"[MeSH Terms] OR ("PFNA"[All Fields] OR (("proximal"[All Fields] OR "proximalization"[All Fields] OR "proximalize"[All Fields] OR "proximalized"[All Fields] OR "proximalizes"[All Fields] OR "proximalizing"[All Fields] OR "proximally"[All Fields] OR "proximals"[All Fields]) AND ("femor"[All Fields] OR "femorals"[All Fields] OR "femur"[MeSH Terms] OR "femur"[All Fields] OR "femoral"[All Fields]) AND ("nails"[MeSH Terms] OR "nails"[All Fields] OR "nail"[All Fields]) AND "anti-rotation"[All Fields]) OR "IMN"[All Fields] OR ("fracture fixation, intramedullary"[MeSH Terms] OR ("fracture"[All Fields] AND "fixation"[All Fields] AND "intramedullary"[All Fields]) OR "intramedullary fracture fixation"[All Fields] OR ("intramedullary"[All Fields] AND "nailing"[All Fields]) OR "intramedullary nailing"[All Fields])) OR ("injections, intravenous"[MeSH Terms] OR (("intraveneous"[All Fields] OR "intraveneously"[All Fields] OR "intravenous"[All Fields] OR "intravenously"[All Fields]) AND ("applicabilities"[All Fields] OR "applicability"[All Fields] OR "application"[All Fields] OR "applications"[All Fields] OR "applicative"[All Fields]))))

Embase

#1. 'intertrochanteric fracture':ti,ab,kw OR 'trochanteric fracture':ti,ab,kw OR 'hip fracture':ti,ab,kw
#2. 'tranexamic acid':ti,ab,kw OR txa:ti,ab,kw
#3. 'intramedullary nail':ti,ab,kw OR imn:ti,ab,kw OR 'proximal femoral nail anti-rotation':ti,ab,kw OR pfna:ti,ab,kw
#4. intravenous:ti,ab,kw
#5. #3 OR #4
#6. #1 AND #2 AND #5

Cochrane library

#1 MeSH descriptor: [Hip Fractures] explode all trees
#2 MeSH descriptor: [Fracture Fixation, Intramedullary] explode all trees
#3 MeSH descriptor: [Tranexamic Acid] explode all trees
#4 MeSH descriptor: [Administration, Intravenous] explode all trees
#5 (intertrochanteric fracture):ti,ab,kw OR (trochanteric fracture):ti,ab,kw OR (hip fracture):ti,ab,kw (Word variations have been searched)
#6 (tranexamic acid):ti,ab,kw OR (TXA):ti,ab,kw (Word variations have been searched)
#7 (intramedullary nail):ti,ab,kw OR (IMN):ti,ab,kw OR (proximal femoral nail anti-rotation):ti,ab,kw OR (PFNA):ti,ab,kw (Word variations have been searched)
#8 (Intravenous):ti,ab,kw (Word variations have been searched)
#9 #1 OR #5
#10 #2 OR #7
#11 #3 OR #6
#12 #4 OR #8
#13 #10 OR #12
#14 #9 AND #11 AND #13
